# Supplementary material for: The causal relationship between the human gut microbiota and pyogenic arthritis: a Mendelian randomization study
Source: Front Cell Infect Microbiol. 2024 Nov 26;14:1452480. doi: 10.3389/fcimb.2024.1452480 (PMC11629706; doi:10.3389/fcimb.2024.1452480)
Supplement: Supplementary file 1 [file DataSheet1.zip › mendelian test/ebi-a-GCST90027589.csv_leave_one_out.pdf]

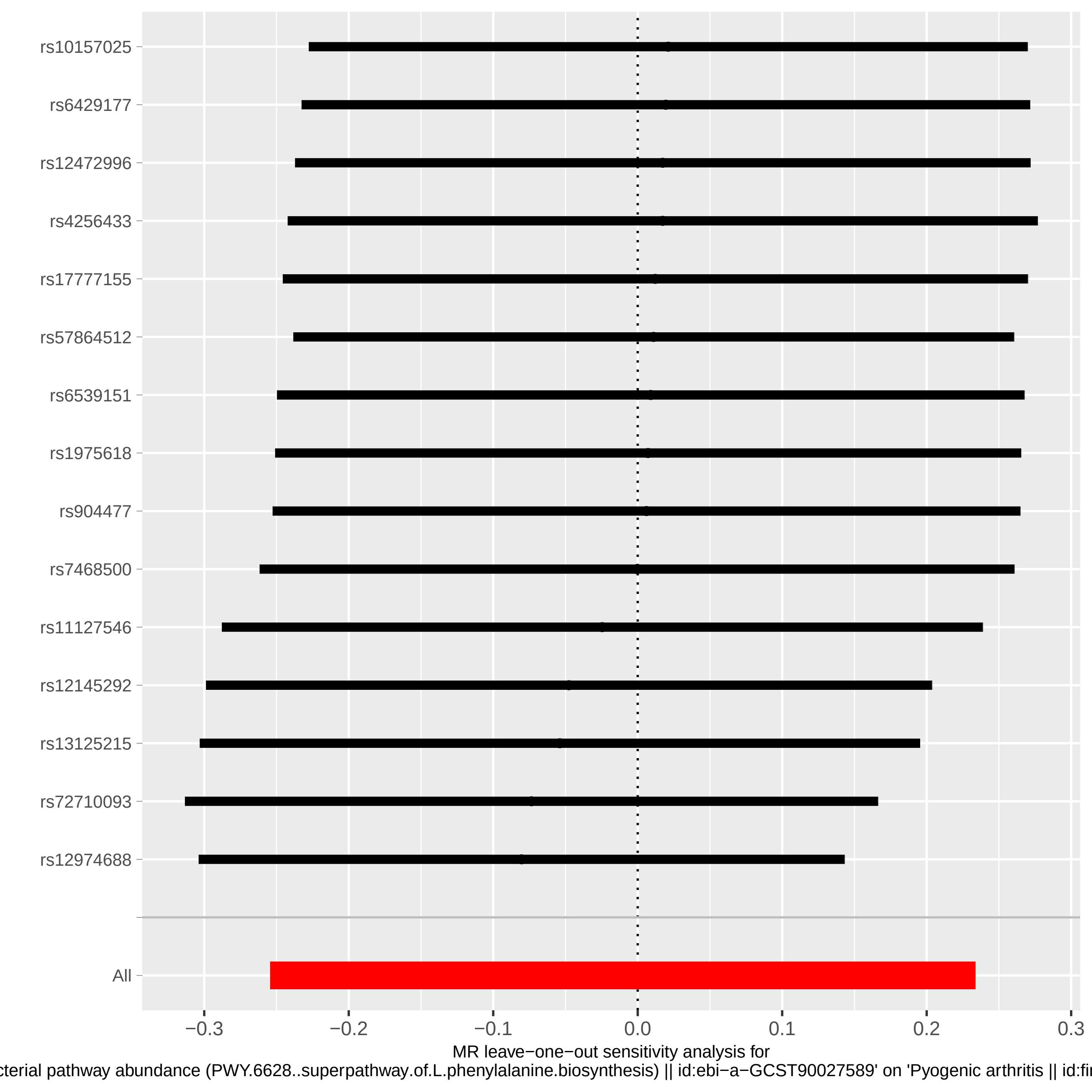

rs10157025
rs6429177
rs12472996
rs4256433
rs17777155
rs57864512
rs6539151
rs1975618
rs904477
rs7468500
rs11127546
rs12145292
rs13125215
rs72710093
rs12974688
All
−0.3
−0.2
−0.1
0.0
0.1
0.2
0.3
MR leave−one−out sensitivity analysis for
cterial pathway abundance (PWY.6628..superpathway.of.L.phenylalanine.biosynthesis) || id:ebi−a−GCST90027589' on 'Pyogenic arthritis || id:fi
